# Arabidopsis GLYI4 Reveals Intriguing Insights into the JA Signaling Pathway and Plant Defense

**DOI:** 10.3390/ijms252212162

**Published:** 2024-11-13

**Authors:** Gaia Salvatore Falconieri, Laura Bertini, Matteo Fiaschetti, Elisabetta Bizzarri, Ivan Baccelli, Carla Caruso, Silvia Proietti

**Affiliations:** 1 Department of Ecological and Biological Sciences, University of Tuscia, Largo dell’Università snc, 01100 Viterbo, Italy; gfalconieri@unitus.it (G.S.F.); lbertini@unitus.it (L.B.); matteo.fiaschetti@studenti.unitus.it (M.F.); elisabetta.bizzarri@unitus.it (E.B.); 2 Institute for Sustainable Plant Protection, National Research Council of Italy, Sesto Fiorentino, 50019 Florence, Italy; ivan.baccelli@ipsp.cnr.it; 3 Institute for Sustainable Plant Protection, National Research Council of Italy, Strada delle Cacce, 73, 10135 Torino, Italy

**Keywords:** Arabidopsis, GLYI4, jasmonic acid, methylglyoxal, plant growth, plant defense, oxidative stress

## Abstract

Plant hormones play a central role in various physiological functions and mediate defense responses against (a)biotic stresses. Jasmonic acid (JA) has emerged as one of the key phytohormones involved in the response to necrotrophic pathogens. Under stressful conditions, plants can also produce small molecules, such as methylglyoxal (MG), a cytotoxic aldehyde. The enzymes glyoxalase I (GLYI) and glyoxalase II primarily detoxify MG. In *Arabidopsis thaliana*, GLYI4 has been recently characterized as having a crucial role in MG detoxification and emerging involvement in the JA pathway. Here, we investigated the impact of a GLYI4 loss-of-function on the Arabidopsis JA pathway and how MG affects it. The results showed that the *glyI4* mutant plant had stunted growth, a smaller rosette diameter, reduced leaf size, and an altered pigment concentration. A gene expression analysis of the JA marker genes showed significant changes in the JA biosynthetic and signaling pathway genes in the *glyI4* mutant. Disease resistance bioassays against the necrotroph *Botrytis cinerea* revealed altered patterns in the *glyI4* mutant, likely due to increased oxidative stress. The MG effect has a further negative impact on plant performance. Collectively, these results contribute to clarifying the intricate interconnections between the GLYI4, MG, and JA pathways, opening up new avenues for further explorations of the intricate molecular mechanisms controlling plant stress responses.

## 1. Introduction

In their habitats, plants constantly face biotic and abiotic stresses, which can negatively influence their development, growth, and reproduction, and, in extreme cases, lead to death. Upon sensing a stimulus, plants initiate a series of morphological, physiological, and biochemical changes, leading to effective defense responses and increased tolerance [1,2]. These finely tuned defense mechanisms are orchestrated by small molecules with hormonal activity, known as phytohormones, which are chemical messengers that are involved in several cellular processes. They are capable of exerting a strong effect on plant metabolic pathways, tissue growth, development regulation, and plant defense. Salicylic acid (SA) and jasmonic acid (JA) and its derivatives, collectively known as jasmonates, are recognized as major defense hormones and have been well characterized over the years [3,4,5]. In particular, SA is primarily associated with the defense against biotrophic pathogens, while JA is primarily linked to the defense against necrotrophic pathogens and herbivores. Auxins, ethylene, abscisic acid, gibberellins, cytokinins, brassinosteroids, and strigolactones have also been reported to play a role as modulators of a plant’s immune signaling network [3]. Many defense strategies are regulated by multiple phytohormones, which often trigger several processes simultaneously. Interactions between different hormone signal transduction pathways, known as cross-talk, add an additional layer of regulation through their antagonistic or synergistic effects [6]. Additionally, plants under stress have the capability to synthesize molecules that, depending on their concentration, can either exhibit toxicity or function as signaling molecules. For instance, under stressful conditions and in response to alterations in their primary metabolism, plants can synthesize toxic aldehydes like methylglyoxal (MG) [7,8,9]. MG is an alpha-oxoaldehyde compound, known for its high reactivity and cytotoxicity, generated as a byproduct of various metabolic processes such as glycolysis, protein degradation, lipid peroxidation, and photosynthesis [10,11]. This compound elicits stomatal closure by triggering the accumulation of reactive oxygen species (ROS) in guard cells through the Ca^2+^-dependent pathway. Furthermore, at elevated concentrations, MG is detrimental to the cell, as it interacts with crucial macromolecules like DNA, RNA, proteins, and lipids, thereby modifying or disrupting their physiological functions, affecting seed germination, plant growth and development, and photosynthesis [7,11,12,13]. The Glyoxalase (GLY) system stands out as the primary detoxification pathway for MG in all organisms, including bacteria, yeast, plants, animals, and humans [11]. The GLY system involves a two-step scavenging process, leading to the formation of D-lactate by two enzyme families: glyoxalase I (GLYI) and glyoxalase II (GLYII) [9,14,15]. Within the annotation of the Arabidopsis (*Arabidopsis thaliana*) genome, there are 11 *GLYI* genes and, among them, *GLYI4* emerges as a pivotal component in the gene network responsible for MG detoxification [16]. A genome-wide association (GWA) study suggested that AtGLYI4 is a novel player in SA-JA hormone signaling and the defense against biotic stresses in Arabidopsis [17,18]. In particular, we previously reported that AtGLYI4 has a crucial impact on MG scavenging, leading to impaired MG detoxification, the accumulation of ROS, stomatal closure, and reduced plant fitness in a *glyI4* loss-of-function mutant [18]. Moreover, the accumulation of MG found in the *glyI4* mutant reduced the efficiency of the JA signaling pathway, increasing the susceptibility of *glyI4* plants to the necrotrophic fungus *Plectospherella cucumerina* [18]. In addition, the impact of *GLYI4*’s loss of function on plant metabolism was investigated in Col-8 and a *glyI4* mutant through a high-resolution mass spectrometry-based metabolomic approach. A pathway enrichment analysis of the identified compounds showed that in the *glyI4* mutant the mechanisms related to plant growth and defense were likely to be affected [19]. Based on the previously observed evidence, in this work we aimed to further investigate the impact of MG on the JA signaling pathway in Arabidopsis in a *glyI4* loss-of-function mutant compared to Col-8. Our work highlighted that the *glyI4* mutant showed a stunted growth, smaller rosette diameter, and reduced leaf size. Additionally, the pigment concentration was altered in the *glyI4* mutant. The analysis of JA biosynthesis and signaling pathway genes demonstrated that JA marker genes are significantly altered in the *glyI4* mutant, further underlining the importance of active GLYI4 for the full functionality of the JA-mediated signaling pathway. Moreover, disease resistance bioassays against the necrotrophic pathogen *Botrytis cinerea* showed an altered pattern in the *glyI4* mutant, and this can be linked to increased oxidative stress. Plant treatment with exogenous MG further corroborated our findings. Taken together, our studies suggest that GLYI4 has a prominent role in maintaining plant health and provide the basis for further investigation into the intricate scenario of plant defense responses.

## 2. Results

### 2.1. The Lack of a Functional GLYI4 Protein Impairs Plant Growth

**Figure 1:**
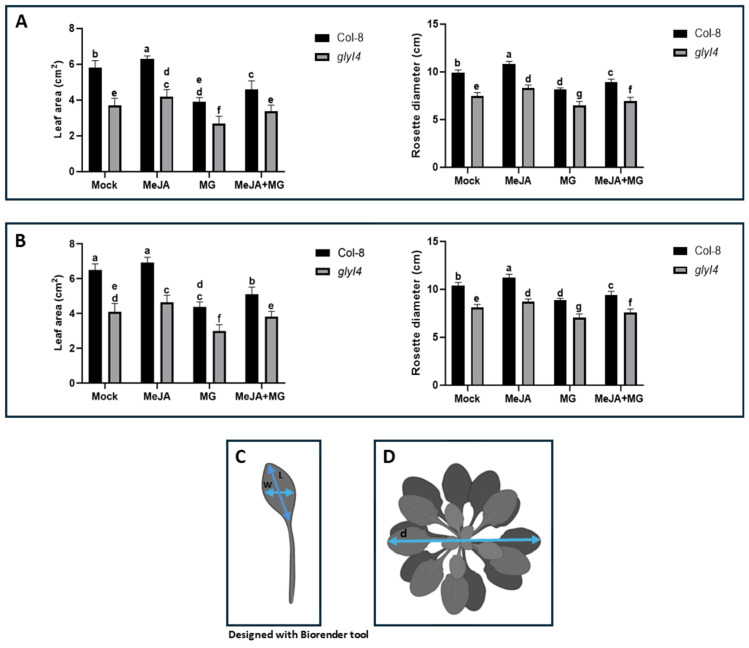
Growth index of Col-8 and *glyI4* treated with Mock, MeJA, MG, and MeJA + MG. Leaf area and rosette diameter 7 (**A**) and 14 days (**B**) after chemical treatments, respectively. Leaf area (LA) (**C**) was calculated based on two treatment-responsive leaves (the 5th and the 11th) using the following formula: LA = L × W, where LA (leaf area); L (leaf length); and W (leaf width). (**D**) The rosette diameter (d) was measured with a ruler. Letters above the bars indicate significant differences between Col-8 and *glyI4* plants among the different treatments (two-way analysis of variance; Šídák’s test; n = 15; *p*-value < 0.0001).

In Arabidopsis, leaf area and rosette diameter measurements are among the most common parameters for assessing plant growth [20]. Here, the effects of Mock (0.015% Silwet L77), MeJA, MG, and MeJA + MG treatments on leaf area and rosette diameter were observed in Col-8 and *glyI4* 7 and 14 days after treatments (Figure 1). Representative photos of the rosette of Col-8 and *glyI4* 7 and 14 days after these treatments are shown in Figure S1.

In general, *glyI4* showed an overall reduction in its leaf area and rosette diameter compared to Col-8 both 7 and 14 days after treatments. In particular, the application of exogenous MeJA generally improved plant growth in both Col-8 and *glyI4* at any time (Figure 1A,B) compared to the Mock, whereas the MG treatment produced the opposite effect in all samples at any time. Interestingly, the combined MeJA + MG treatment showed, in both Col-8 and *glyI4*, that MeJA is able to slightly attenuate the negative effect of MG on plant growth.

### 2.2. GLYI4 Influences Plant Pigment Content

**Figure 2:**
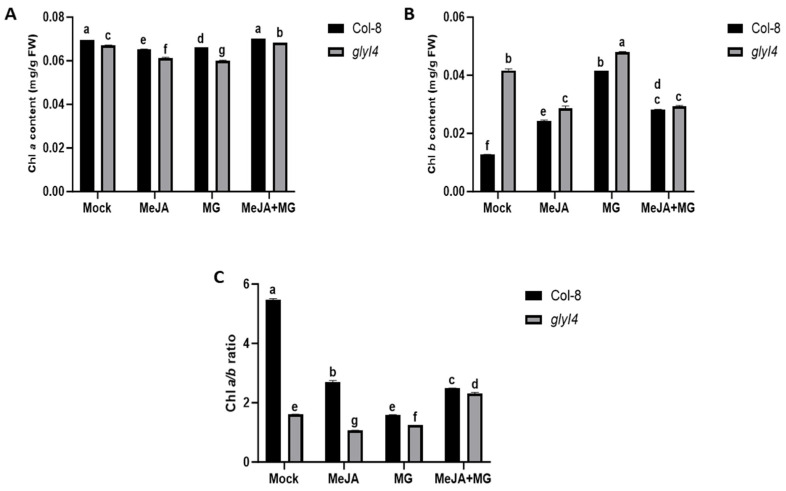
Chlorophyll content in Col-8 and *glyI4* treated with Mock, MeJA, MG, and MeJA + MG. (**A**) Chlorophyll *a* (Chl *a*) content; (**B**) chlorophyll *b* (Chl *b*) content; (**C**) chlorophyll *a**/**b* ratio. Letters above the bars indicate significant differences between Col-8 and *glyI4* among the treatments (two-way analysis of variance; Šídák’s test; n = 3; *p*-value < 0.0001).

Photosynthetic parameters are generally used as indicators of plant responses to stress [21,22]. Here, we investigated the chlorophyll content after Mock, MeJA, MG, and MeJA + MG treatments. As shown in Figure 2A, the content of Chlorophyll *a* (Chl *a*) significantly diminished in *glyI4* compared to Col-8 over all the treatments; in contrast, the content of Chlorophyll *b* (Chl *b*) significantly increased in all treated *glyI4* samples, compared to Col-8, with the exception of the double treatment (Figure 2B). It is noteworthy that MG is the most efficient treatment at increasing the Chl *b* content, both in Col-8 and *glyI4*. Additionally, as shown in Figure 2C, the Chl *a*/*b* ratio, which represents the trade-off efficiency between light capture and light conversion, was significantly lower in all *glyI4* samples than in Col-8 ones.

**Figure 3:**
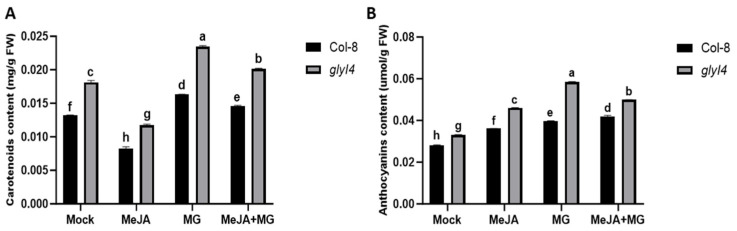
Carotenoid and anthocyanin contents in Col-8 and *glyI4* treated with Mock, MeJA, MG, and MeJA + MG. (**A**) Carotenoid content; (**B**) anthocyanin content. Letters above the bars indicate significant differences between Col-8 and *glyI4* among the different treatments (two-way analysis of variance; Šídák’s test; n = 3; *p*-value < 0.0001).

To obtain a better overview of the plant response to different treatments, we also analyzed the content of protective pigments, i.e., carotenoids and anthocyanins, since they are known to play a key role in the response to stress [23,24] (Figure 3A,B).

Both pigments showed a statistically significant increase in *glyI4* compared to Col-8, either in the Mock treatment or after treatment with chemicals. In particular, the MG treatment induced the strongest increase in the carotenoid and anthocyanin contents in *glyI4* compared to other treatments. In *glyI4*, the combined MeJA + MG treatment caused a significant reduction in the pigment content compared to MG, indicating a possible contribution of MeJA to the attenuation of the stress effect induced by the MG treatment. These findings suggest that the *glyI4* mutant exhibits impaired pigment machinery and that MG may play a prominent role in that.

### 2.3. GLYI4 Involvement in JA Signaling Pathway

**Figure 4:**
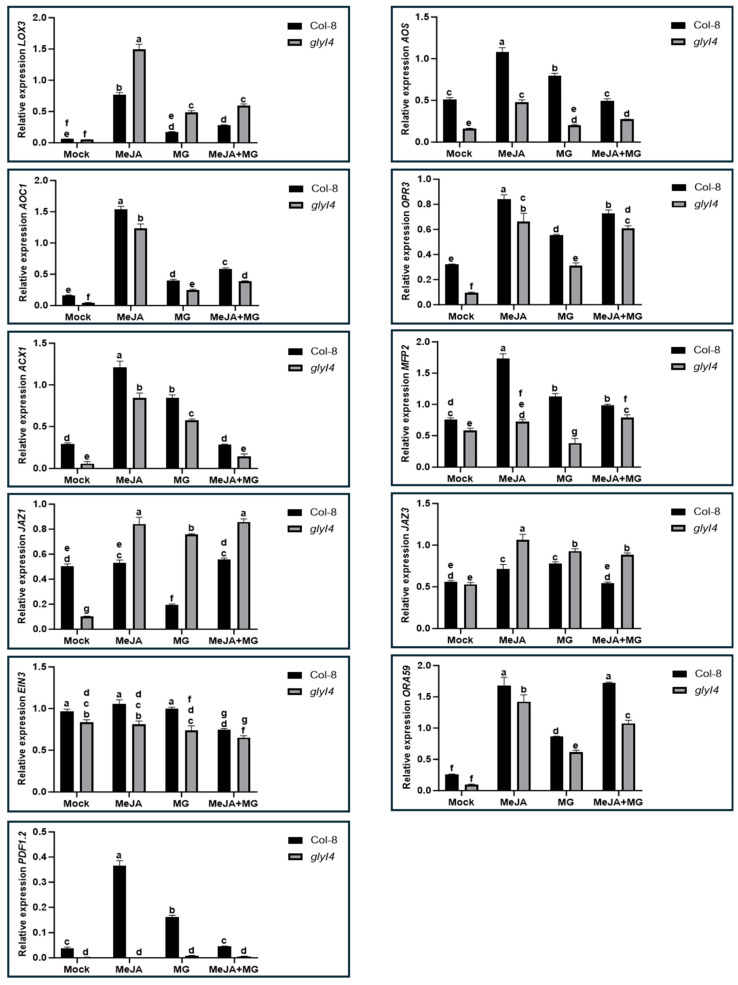
Transcript levels of JA marker genes in Col-8 and *glyI4* treated with Mock, MeJA, MG, and MeJA + MG. Expression analyses were performed 24 h after the chemical treatment of 5-week-old plants. The expression is relative to the reference gene *PP2AA3.* Error bars represent standard deviation. Letters above the bars indicate significant differences between Col-8 and *glyI4* plants among the different treatments (two-way analysis of variance; Šídák’s test; n = 3; *p*-value < 0.0001).

To generate findings on the impact of GLYI4 on the defense mechanisms of Arabidopsis, we conducted a gene expression analysis, pinpointing a set of markers involved in the JA biosynthetic pathway and JA signaling in both Col-8 and *glyI4* 5-week-old plants after Mock, MeJA, MG, and MeJA + MG treatments. The first group included lipoxygenase 3 (*LOX3*), allene oxide synthase (*AOS*), allene oxide cyclase 1 (*AOC1*), 12-oxophytodienoate reductase 3 (*OPR3*), acyl-CoA oxidase 1 (*ACX1*), and multifunctional protein 2 (*MFP2*) and is associated with JA biosynthesis, which occurs in the cytoplasm, peroxisomes, and chloroplasts. LOX3 oxygenates fatty acids, producing hydroperoxides, which are converted by AOS into allene oxide; AOC1 catalyzes the cyclization of allene oxide and paves the way for OPR3 to transform the precursor oxophytodienoic acid (OPDA) into its JA biologically active form. ACX1 and MFP2 contribute to peroxisomal metabolism, aiding in the breakdown of lipids and providing substrates for JA synthesis. The second group includes genes involved in JA signal transduction within the nucleus. In basal conditions, some of these markers, like the Jasmonate ZIM-domain (JAZ), act as co-receptors of jasmonate-isoleucine (JA-Ile), its JA bioactive form. JAZ proteins, including JAZ1 and JAZ3, act as repressors in the absence of JA, binding to transcription factors and preventing the activation of JA-responsive genes [25,26]. Under stress conditions, when JA-Ile is perceived, these JAZ proteins are degraded and, simultaneously, transcription factors, such as Ethylene-Insensitive 3 (EIN3), traditionally associated with ethylene (ET) signaling, are released to activate the expression of downstream genes [26]. Some of these downstream genes encode for other transcription factors, like octadecanoid-derivative responsive Arabidopsis 59 (ORA59), which in turn regulates the expression of downstream defense-related genes such as *PDF1.2*, one of the main marker genes of the ERF branch of JA signaling. Figure 4 displays the results of a qRT-PCR analysis of all the examined genes.

In *glyI4* mutants, an overall reduction in the expression of most of the analyzed genes, regardless of the treatment applied, can be observed. In particular, in *glyI4*, the expression of the *AOS*, *AOC1*, *OPR3*, *ACX1*, *MPF2*, *EIN3*, *ORA59,* and *PDF1.2* genes decreased after the MeJA treatment compared to in Col-8; the MG treatment had an effect, further lowering the expression of the genes listed above (with the exception of *EIN3*). The combined MeJA + MG treatment significantly attenuated the MG-induced downregulation of some of the abovementioned genes (*AOC1*, *OPR3*, *MFP2*, *ORA59*). A different trend in gene expression was followed by *LOX3* and by the *JAZ* repressors (*JAZ1* and *JAZ3*), which were significantly upregulated in *glyI4* compared to Col-8 after chemical treatments. In general, the observed difference in gene expression between *glyI4* and Col-8 may be attributed to the increased endogenous MG content in the mutant, as previously reported [18], which could potentially disrupt the JA signaling response.

### 2.4. GLYI4 Affects Plant Susceptibility to the Fungal Necrotrophic Pathogen Botrytis Cinerea

**Figure 5:**
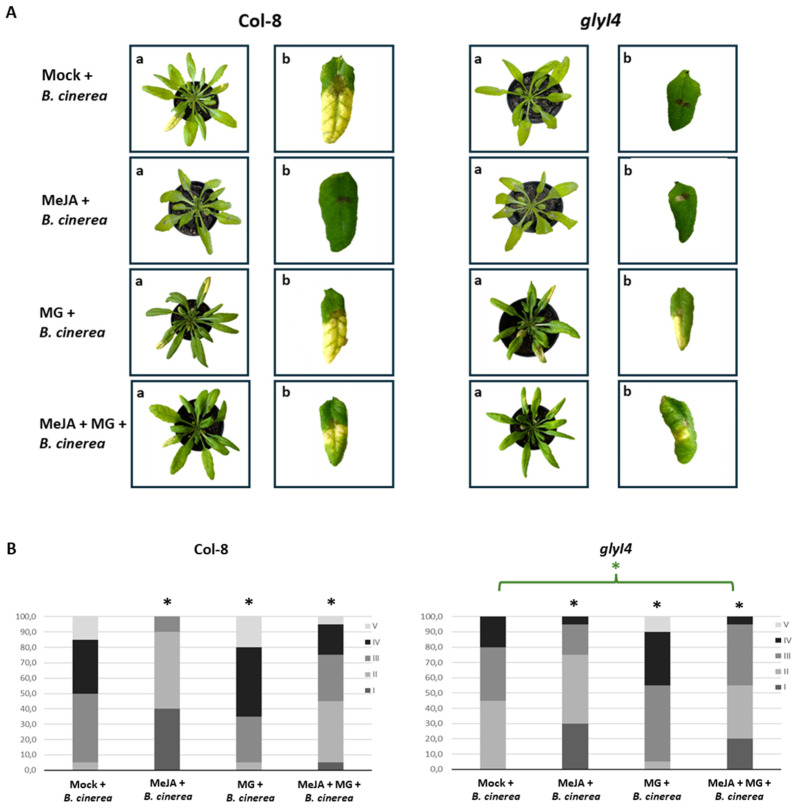
*Botrytis cinerea* resistance bioassay in Col-8 and *glyI4* treated with Mock, MeJA, MG, and MeJA + MG. (**A**) Representative photographs of *B. cinerea* disease symptoms in Col-8 and *glyI4* mutant plants 3 days after inoculation. (a) General overview of the plant after infection; (b) focus on a single leaf affected by disease symptoms. (**B**) Distribution of disease symptoms in Col-8 and *glyI4*. The bars indicate the frequency distribution (in %) of disease symptoms. The evaluation of this disease is based on the diameter of the lesion on the leaves and includes 5 classes: I, lesion of 2 mm; II, lesion of 2 mm + chlorosis; III, lesion between 2 and 4 mm + chlorosis; IV, lesion larger than 4 mm + chlorosis; V: widespread leaf necrosis. A black asterisk above the bars indicates that there is a statistically significant difference between Mock + *B. cinerea* and each treatment (χ^2^ test, n = 9; *p*-value < 0.05). The asterisk above the bracket indicates significant differences in the response to any treatment between *glyI4* and Col-8.

Necrotrophic pathogens are generally deterred by defenses controlled by the JA and ET pathways. In order to investigate the influence of GLYI4 in the susceptibility to *B. cinerea* of Col-8 and *glyI4* plants, a disease resistance bioassay was performed in the presence of the Mock, MeJA, MG, and MeJA + MG treatments. The symptoms were evaluated three days after the inoculation of *B. cinerea* spores and representative images are shown in Figure 5A.

**Figure 6:**
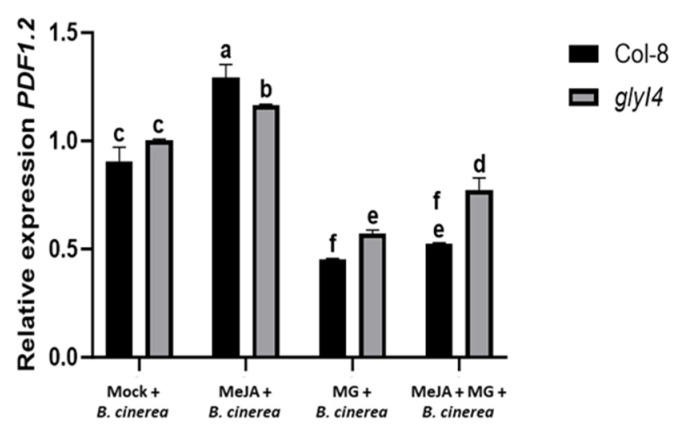
*PDF1.2* transcript levels in Col-8 and *glyI4* treated with Mock, MeJA, MG, and MeJA + MG and infected with *Botrytis cinerea*. The analysis was carried out 3 days after inoculation. Error bars represent standard deviation. The expression is relative to *PP2AA3.* Letters above the bars indicate significant differences between Col-8 and *glyI4* among the different treatments (two-way analysis of variance; Šídák’s test; n = 3; *p* value < 0.0001).

The disease symptoms were classified into five classes, ranging from I (lesion of 2 mm) to V (widespread leaf necrosis) (Figure 5B). The overall disease symptom score showed a different outcome depending on the genotype and the applied treatment. In the Mock treatment, *glyI4* showed increased resistance to *B. cinerea* compared to Col-8, as previously observed in [17]. Within each genotype, we observed that MeJA-treated plants had less severe symptoms due to the *B. cinerea* infection compared to MG and MeJA + MG; the MG treatment showed the worst conditions of all treatments; in the MeJA + MG treatment, an increased resistance compared to that of the Mock treatment was observed, but lower than that of plants treated with MeJA only. As for the comparison between the two genotypes, we found that in *glyI4,* MeJA is less effective in protecting plants from the fungal pathogen compared to Col-8, giving more proof of the perturbation of the JA pathway in the mutant. It is noteworthy that the chemical treatments did not have any significant effect on the fungal growth of *B. cinerea* itself (Figure S2), so the different leaf symptoms reflected the particular response of the plant to each applied treatment. As a further investigation, the expression of the JA marker gene *PDF1.2* in Col-8 and *glyI4* after a *B. cinerea* infection and following chemical treatments was evaluated by qRT-PCR (Figure 6).

**Figure 7:**
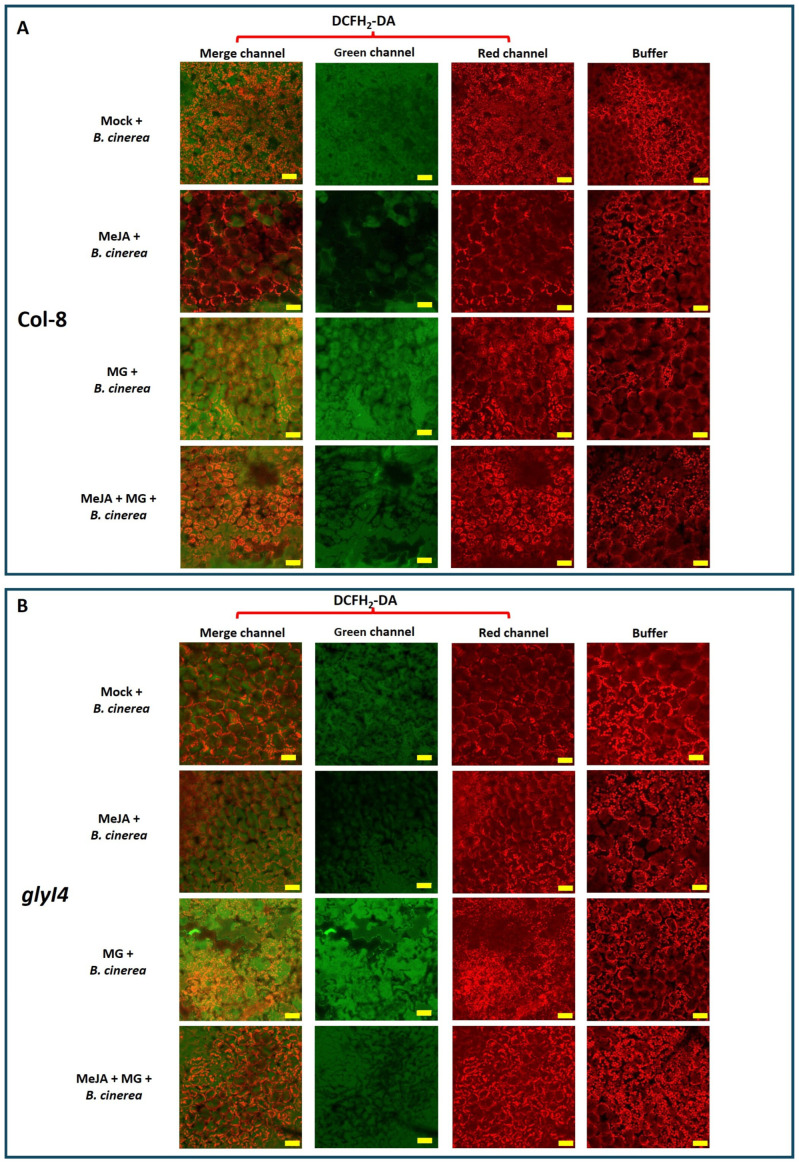
Detection of ROS in Col-8 (**A**) and *glyI4* (**B**) leaves after Mock, MeJA, MG, and MeJA + MG treatments and *Botrytis cinerea* infection. The detection of ROS was carried out by using DCFH_2_-DA or buffer (negative technical control). Fluorescence was observed under an LSM 710 confocal microscope with a Plan Neofluar 20/1.30 objective. Laser excitation lines were used, i.e., 488 for probe detection (green) and 561 nm for chlorophyll auto-fluorescence (red). The bar corresponds to 50 μm. The merged-, green-, and red-channel images are shown.

The results showed that both genotypes followed the same expression pattern. In particular, the highest *PDF1.2* expression was found in MeJA-treated plants and the lowest in MG-treated plants, while the combined MeJA + MG treatment partially ameliorated the decrease in *PDF1.2* expression induced by the MG treatment, and significantly so in *glyI4*. Finally, to evaluate *B. cinerea*-induced oxidative stress, reactive oxygen species (ROS) levels were detected in Col-8 and the *glyI4* mutants after *B. cinerea* infection following Mock, MeJA, MG, and MeJA + MG treatments using 2′,7′-dichlorofluorescein diacetate (DCFH_2_-DA) and confocal microscope observation. The oxidation of the dye induced by ROS produces green fluorescence, the intensity of which is used as an index of oxidative stress. Leaves treated with the buffer (Tris-HCl 10 mM), which were used as a technical control, showed only red fluorescence due to the chlorophyll absorbance spectrum, while all samples treated with DCFH_2_-DA showed very strong green fluorescence, highlighting the presence of high ROS levels, although at different levels (Figure 7).

**Figure 8:**
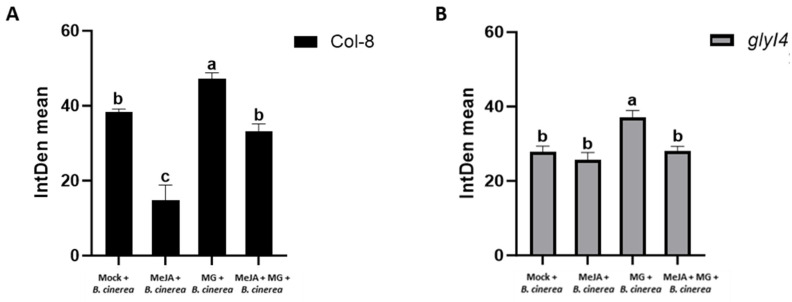
Quantification of green fluorescence detected using DCFH_2_-DA in Col-8 (**A**) and *glyI4* (**B**) after Mock, MeJA, MG, MeJA + MG treatments and *Botrytis cinerea* infection. The integrated density mean is reported. Letters above the brackets indicate a statistically significant difference between samples (one-way analysis of variance; Tukey’s test; n = 3; *p*-value < 0.0001). Quantification has been performed using ImageJ, version 1.52a.

To obtain a quantitative comparison and appreciate the differences between samples, the quantification of green fluorescence was reported as an integrated density mean using a measurement tool within ImageJ software (version 1.52a) for both Col-8 and *glyI4* (Figure 8).

Interestingly, the application of MG caused a statistically significant increase in green fluorescence, indicating a heightened oxidation level in both Col-8 and *glyI4*. It is noteworthy that after the MeJA treatment, the green fluorescence was reduced, although only significantly so in Col-8. In *glyI4,* this effect was less apparent, likely due to a defective perception and/or signaling of MeJA. These findings align with the outcomes observed in the disease bioassay presented earlier.

To obtain a quantitative comparison and appreciate the differences between samples, the quantification of green fluorescence was reported as an integrated density mean using a measurement tool from ImageJ software for both Col-8 and *glyI4* (Figure 8).

## 3. Discussion

Plant defense responses are mainly regulated by phytohormones. Through the cross-communication of their signaling pathways, plants have a vast regulatory potential that allows them to rapidly adapt to biotic and abiotic stressors, ensuring cost-efficient responses and ultimately enhancing their fitness [1,3]. In Arabidopsis, the JA signaling pathway has been extensively studied over the past few decades, and the role of novel players is of great interest. In this work, we aimed to add to the findings on the novel JA novel GLYI4, an enzyme belonging to the Glyoxalase I (GLYI) family.

Firstly, we used *glyI4* mutant plants to investigate the impact of GLYI4 on plant growth and development, and investigated this in the presence of MeJA, MG, and MeJA + MG treatments as well, demonstrating that the lack of function of this enzyme leads to stunted growth in mutant plants, regardless of the treatment, compared to Col-8 (Figure 1). This trend was further confirmed to be especially heightened in the presence of the MG treatment. This is consistent with previous observations in which higher endogenous levels of MG strongly impaired plant growth and development, leading to reduced plant fitness [18,27]. In fact, Arabidopsis mutants deficient in glyoxalase enzymes showed stunted growth, a smaller rosette diameter, and reduced leaf sizes, which are linked to increased MG toxicity, which causes oxidative stress, protein modification, and damage to cell structures [28,29]. GLY family mutants also show premature leaf senescence and increased leaf yellowing due to their inability to detoxify MG, disrupting normal cellular function [28,30,31]. In our study, the stress phenotype observed in *glyI4* plants treated with MG was attenuated by the MeJA + MG treatment, likely due to the contribution of MeJA working against the detrimental effect of MG accumulation. Indeed, different studies have demonstrated that MeJA has a restoring effect on plant morphometric parameters in different plant species, such as Arabidopsis, Artemisia (*Artemisia annua* L.), sweet basil (*Ocimum basilicum* L.), and peppermint (*Mentha piperita* L.), after different stress signals like water stress or artificial wounding [32,33,34,35].

As has been widely reported, photosynthetic parameters are other important markers of plant stress responses [21,36]. In fact, photosynthesis can be negatively impacted by exposure to a variety of abiotic and biotic stresses. Here, we showed that Chl *a* is downregulated in *glyI4* compared to Col-8, regardless of the treatment, although the MG treatment had the most severe impact on *glyI4* (Figure 2A). In contrast, the content of Chl *b* was upregulated in *glyI4* mutant plants compared to Col-8 in all conditions except MeJA + MG (Figure 2B). Chl *a* is the energy transduction core of the light harvesting system and is often downregulated in stressful conditions [37]. The regulation of Chl *b* in plants subjected to stress is more complex and less well documented, since its response to stress can be different from that of Chl *a*. In fact, different studies have demonstrated that plants may upregulate Chl *b* as a compensatory mechanism to favor photosynthetic apparatus adaptation [37,38]. The production of protective pigments and antioxidants is also part of plants’ broad stress response mechanism. Carotenoids and anthocyanins are crucial pigments within the photosynthetic machinery of plants, and they have a variety of functions like antioxidant and light-damage prevention in the non-photosynthetic parts of a plant [39]. Therefore, they can also be found in high concentrations in stressed samples. In our study, we found that both pigments were higher in the *glyI4* mutant compared to Col-8, especially after the MG treatment (Figure 3A,B). Indeed, different studies have demonstrated that the accumulation of carotenoids and anthocyanins in MG-treated plants indicates a plant resistance mechanism deployed to protect the plant from MG-induced damages [40,41]. These results confirm the importance of these compounds as antioxidant and protective molecules, highlighting their crucial role in stress conditions in different plant species [29,39,42].

Our previous study suggested that JA signaling appears to be compromised in the *glyI4* mutant [18]. Here, we aimed to investigate the expression of a set of JA marker genes, from biosynthesis to signaling, in Col-8 and *glyI4* plants after MeJA, MG, and MeJA + MG treatments to understand at which stage GLYI4 could impact the JA signaling pathway. Our work showed that the *LOX3* gene has a different trend compared to the other analyzed genes, highlighting that there was an upregulation of this gene in the mutant *glyI4* plant’s in response to the chemical treatments. LOX3 is involved in the first step of JA production and is responsible for the oxygenation of polyunsaturated fatty acids like linolenic acid [43], while also taking part in lipid peroxidation in different plant species, as has been widely reported [44,45,46,47]. In fact, under biotic or abiotic stress, the balance between protective signaling and cellular damage can tip toward the latter, causing lipid peroxidation that can lead to membrane breakdown, the loss of cellular compartmentalization, and potentially cell death [48]. Recently, we demonstrated that the *glyI4* mutant suffers from lipid peroxidation, probably due to increased MG accumulation [19], leading to the hypothesis that the increase in this toxic compound experienced by mutant plants has an impact on the upregulation of the *LOX3* gene. In our study, the qRT-PCR analysis revealed that the transcriptional repressors JAZ1 and JAZ3 have a different trend than that of the other genes considered. In particular, they are upregulated compared to the others, which leads us to the assumption that MG accumulation could have an impact on their expression. In fact, while MG may initially promote JA signaling by degrading JAZ proteins, prolonged or excessive stress could lead to feedback mechanisms where JAZ proteins are upregulated to balance the activation of JA-responsive genes. Recently, it was demonstrated that the two catabolic pathways of JA contribute differentially to JA-Ile removal depending on distinct leaf stresses and that further variations could be expected in other organs or under other environmental constraints [49]. Moreover, a gene expression study of JAZ repressors in sweet potato (*Ipomoea batatas* L.) revealed that the levels of some JAZ repressors were significantly different under different stresses [50].

In addition, we investigated how chemical treatments influence Col-8 and *glyI4* plants’ susceptibility to *B. cinerea*. Firstly, we showed that in the Mock condition, *glyI4* is more resistant to this necrotrophic pathogen compared to Col-8. This evidence corroborates the findings in Proietti et al., 2018, where GLYI4 emerged as a positive regulator of SA-mediated antagonism of the JA pathway [17]. In fact, the latter study demonstrated how, in *glyI4,* the lack of SA-mediated antagonism of the JA pathway was associated with an enhanced level of resistance against *B. cinerea* [17]. Besides this, we also found that the MeJA treatment is less effective in protecting *glyI4* from disease compared to Col-8 (Figure 5A,B), as confirmed by the downregulation of *PDF1.2* expression, often considered a marker gene for *B. cinerea* infection (Figure 6). Finally, infection with *B. cinerea* induced a significant change in the oxidative state of the plants, leading to elevated levels of ROS in both Col-8 and *glyI4*, although to a different magnitude between genotypes and among treatments (Figure 7 and Figure 8). It is interesting to note that the MeJA treatment was less effective at mitigating oxidative stress in *glyI4* compared to Col-8. Proietti et al. (2019) [18] demonstrated that the *glyI4* mutant suffers from high levels of ROS, probably due to MG accumulation. Moreover, oxidative stress is a major cause of reduced photochemical activity in plants, primarily through the generation of ROS, which can damage cellular components, including those involved in photosynthesis, or disrupt the electron transport chain and compromise photosynthetic enzymes [51]. These could also explain the impairment of photosynthetic pigments found in our study.

**Figure 9:**
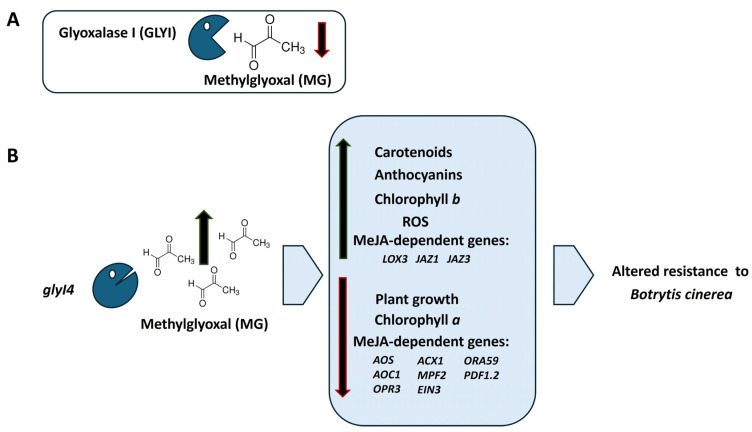
Summary of the main results obtained. (**A**) The GlyI enzyme family is involved in MG scavenging. (**B**) In *glyI4* mutant plants, characterized by defective MG scavenging, higher carotenoid, anthocyanin, and chlorophyll *b* contents; higher ROS production; and an up-regulation of the MeJA-dependent *LOX3*, *JAZ1*, *JAZ3* genes are observed. On the contrary, plant growth and chlorophyll ***a*** content are compromised. Additionally, a set of MeJA-dependent genes such as *AOS*, *AOC1*, *OPR3*, *ACX1*, *MPF2*, *EIN3*, *ORA59*, and *PDF1.2* are downregulated. Finally, *glyI4* mutant plants show altered resistance to the necrotrophic fungal pathogen *Botrytis cinerea*.

All in all, this study investigates the impact of GLYI4’s loss of function on the Arabidopsis JA signaling pathway and how MG affects it. Our results show that the *glyI4* mutant leads to stunted growth, a smaller rosette diameter, reduced leaf sizes, and impaired pigment concentrations. The analysis of JA biosynthesis and signaling pathway genes also revealed significant changes in the JA marker genes. Disease resistance bioassays revealed altered patterns in the *glyI4* mutant, likely linked to increased oxidative stress. Under the effect of MG, plant performance was further negatively affected. Collectively, these results contribute to elucidating the tangled interconnections between GLYI4, MG, and the JA signaling pathway, revealing findings that corroborate previous results [17,18]. An overview of our main findings is shown in Figure 9.

To conclude, our results enrich our understanding of plant defense strategies and also provide crucial insights into the dual role of MG in disrupting inter-pathway communication while affecting specific defense mechanisms. This research helps fill a critical knowledge gap, but it also sets the stage for further exploration into the molecular complexities governing plant stress responses.

## 4. Materials and Methods

### 4.1. Plant Materials and Growth Conditions

The *A. thaliana* T-DNA line in Col-8 background *glyI4* (AGI: At1g15380) was purchased from NASC (http://arabidopsis.info/, (accessed on 20 August 2014)), available with the ID SALK_067593C. The same mutant has already been tested and used for experiments [17,18,19]. Arabidopsis *glyI4* and Col-8 seeds were sown in cultivation containers filled with autoclaved pot soil. The pot soil was supplemented with Hoagland salt solution (Sigma, Steinheim, Germany) at half strength (1.6 g in 100 mL of dH_2_O), which was used to improve the seeds’ germination. In order to achieve a high relative humidity for germination, the cultivation containers were enclosed in a tray with water and covered with a transparent lid. Seeds were then stratified for 2 days, at 4 °C, in the dark to ensure homogeneous germination, after which the tray was moved to a growth chamber with an 8 h day/16 h night rhythm, a temperature of 24 °C, and a light intensity of 100 $\mu mol\;m^{-2}\;s^{-1}$. Ten-day-old seedlings were transplanted into individual pots containing an autoclaved mixture of river sand and potting soil (1:1 *v*:*v*). Pots were watered from the bottom three times per week. Five-week-old plants were used for all experiments.

### 4.2. Chemical Treatments

Five-week-old Arabidopsis plants (Col-8 and *glyI4*) were treated with MeJA (Serva, Brunschwig Chemie, Amsterdam, The Netherlands), MG (Sigma, Steinheim, Germany), or a combination of MeJA/MG by dipping the plants for about 5 s in a solution containing 100 µM MeJA or 10 mM MG or a combination of 100 µM MeJA/10 mM MG, all with 0.015% (*v*/*v*) Silwet L77 (Van Meeuwen Chemicals BV, Weesp, The Netherlands). MeJA was diluted from a 1000-fold concentrated stock in 96% ethanol. The Mock solution contained 0.015% Silwet L77 only.

### 4.3. Analysis of Growth Parameters

One and two weeks after their treatment with a Mock, MeJA, MG, or MeJA + MG solution, the leaf area and rosette diameter of Col-8 and *glyI4* plants were measured with a ruler. Leaf area was calculated on the fifth and eleventh leaves (both middle-age) using the following formula: LA = L × W, where LA (leaf area); L (leaf length); and W (leaf width). The rosette diameter was calculated by measuring the plant diameter with a ruler one and two weeks after treatment. For each genotype in each independent experiment, we analyzed data from a total of five biological replicates, considering the average leaf area and rosette diameter from three plants as our biological replicate.

### 4.4. Determination of Chlorophyll and Carotenoid Contents

The determination of chlorophyll and carotenoid contents was carried out for Mock-, MeJA-, MG-, and MeJA + MG-treated Col-8 and *glyI4* plants. According to the protocol in [52], 0.15 g of leaf material was homogenized in liquid nitrogen, to which 1.5 mL of 80% acetone was added. The homogenate was centrifuged at 7000 rpm for 3 min and the supernatant was recovered while the pellet was resuspended in 80% acetone (0.5 to 1 mL). The centrifugation and acetone suspension steps were repeated until complete decolorization of the supernatant. The final supernatant volume of each sample was reported. These data were used to calculate the concentration of the chlorophylls and carotenoids in the starting plant material. Spectrophotometer readings were taken with quartz cuvettes at the following absorbances:

- 663 nm: preferential absorption of chlorophyll *a*.
- 648 nm: preferential absorption of chlorophyll *b*.
- 470 nm: preferential absorption of carotenoids.

The concentration of chlorophylls and carotenoids was calculated as follows:

Chl *a* = 12.25 × ABS_663_ − 2.55 × ABS_648_ (µg/mL)

Chl *b* = 20.31 × ABS_648_ − 4.91 × ABS_663_ (µg/mL)

Car = (1000 × ABS_470_ − 1.82 × [chl *a*]) − (85.02 × [chl *b*])/198 (µg/mL).

Then, to obtain a concentration expressed in mg/g of fresh weight (FW), each value expressed in µg/mL was multiplied by the mL of the recovered supernatant. The obtained mg was divided by the mg of FW. For each genotype, the analysis was conducted using a pool of two leaves (the fifth and the eleventh) from three plants, harvested twenty-four hours after the treatment. Each pool represents a biological replicate. For each independent experiment we analyzed data from three biological replicates.

### 4.5. Determination of Anthocyanin Content

The determination of the anthocyanin content was performed for Mock-, MeJA-, MG-, and MeJA + MG-treated Col-8 and *glyI4* plants. In total, 200 mg of fresh leaf tissue was cold-homogenized and then 2 mL of methanol acidified with 0.1% pure HCl (MeOH:HCl; *v*:*v*) was added. At the end of the extraction, the supernatant was collected in an Eppendorf tube and centrifuged at 10,000 rpm for 10 min. Portions of 100 μL of the supernatant were placed in UV-vis polystyrene cuvettes and then added to 900 μL of the same previously prepared solution of methanol acidified with 0.1% with pure HCl (MeOH:HCl; *v*:*v*). Their maximum absorption was evaluated at 536 nm. The blank was prepared with 1 mL of methanol, acidified as above. The molar extinction coefficient of cyanidin-3-glucoside in the same solvent (30,400 mol^−1^ cm^−1^) was used to calculate the anthocyanin molar concentration. For each genotype, the analysis was conducted using two leaves (the fifth and eleventh) from three plants, harvested twenty-four hours after treatment, which were pooled together. Each pool represents a biological replicate. For each independent experiment we analyzed data from three biological replicates.

### 4.6. RNA Extraction and RT-qPCR

Total RNA was isolated from Col-8 and *glyI4* leaves after their treatment with Mock, MeJA, MG, or MeJA + MG using the “Nucleospin RNA II” kit (Machery-Nagel, Düren, Germany) according to the manufacturer’s instructions. The RNA’s quality and concentration were estimated by agarose gel electrophoresis and spectrophotometric readings, respectively. DNA contamination was assessed by detecting the absence of amplification bands in no-RT samples. cDNA was synthesized using the ImProm-II™ Reverse Transcription System (Promega, Madison, WI, USA), starting with 1 µg of RNA as a template and using oligo-dT primers for first-strand synthesis. Diluted cDNA was used as the template for Real-Time qPCRs with a CFX 96 Touch Real-Time PCR detection system (Bio-Rad, Hercules, CA, USA). Each 10 µL of reaction mix contained 5 µL of Sso Advanced SYBR Green Supermix (Bio-Rad) and 0.5 µM (final concentration) of gene-specific primers. The primers were designed with Primer3 software (http://bioinfo.ut.ee/primer3-0.4.0/, (accessed on 5 December 2022)) under default conditions. The amplification program was as follows: 95 °C for 5 s; 40 cycles at 95 °C for 5 s; and annealing with primers at 60 °C for 45 s. To detect and exclude non-specific amplicons, the melting curves of all PCR products were analyzed (65–95 °C with an increase of 0.5 °C every 5 s). Real-time DNA amplification was processed using CFX Manage^TM^ Software, version 3.1 (Bio-Rad), accessed on 25 July 2024). Transcript levels were calculated relative to the reference gene At1g13320 (*PP2AA3*) [53], using the 2^−∆∆CT^ method, by applying efficiency correction formula [54]. For each genotype, the analysis was conducted using two leaves (the fifth and the eleventh) from three plants, harvested twenty-four hours after treatment, which were pooled together. Each pool represents a biological replicate. For each independent experiment we analyzed data from three biological replicates. The primers used are listed in the Supplementary Material (Table S1).

### 4.7. Disease Resistance Bioassays

*Botrytis cinerea* strain B05.10 [55] was grown for 2 weeks on half-strength potato dextrose agar (PDA; Difco Laboratories, Leeuwarden, The Netherlands) plates containing penicillin (100 μg mL^−1^) and streptomycin (200 μg mL^−1^) at room temperature, as described previously [56]. *B. cinerea* spores were subsequently collected, filtered through glass wool, and resuspended in half-strength potato dextrose broth (PDB; Difco Laboratories, Leeuwarden, The Netherlands) to a final density of 1 × 10^5^ spores mL^−1^ and left for an incubation period of 3 h. Twenty-four hours before infection, 5-week-old Col-8 and *glyI4* plants were subjected to chemical treatments as described in Section 4.2 (Mock, MeJA, MG, MeJA + MG). The *B. cinerea* infection was performed by applying 5 μL droplets of the spore suspension to six leaves of each plant (for each genotype, in each independent experiment, three plants per treatment were inoculated). The plants were placed in a box with a transparent lid to increase the relative humidity to 100% to aid the infection. Three days after the *B. cinerea* inoculation, the lids were removed and the symptoms were scored as one of five disease severity classes: a lesion of 2 mm (Class I); lesion of 2 mm + chlorosis (Class II); lesion between 2 and 4 mm + chlorosis (Class III); lesion larger than 4 mm + chlorosis (Class IV); and widespread leaf necrosis (Class V) [56]. The percentage of leaves in each class was calculated per plant (χ^2^ test; n = 9 plants per treatment). The effect of the chemical treatments on the growth rate of *B. cinerea* was evaluated by applying 500 μL of the solutions around the fungal growth area on 2-week-old plates. The growth rate was calculated four days after the chemical treatment, as [(mean growth area after treatment − mean growth area before treatment)/mean growth area before treatment]. Finally, the growth rate in all samples considered was normalized to that in Mock + *B. cinerea*, which was set as 1. For each genotype and for each independent experiment, three biological replicates (i.e., three plates) per treatment were considered in our analysis.

### 4.8. ROS Detection in Arabidopsis Leaves

ROS detection was performed as previously described [57,58]. Briefly, ROS production was revealed using the specific probe 2′,7′-dichlorofluorescein diacetate (DCFH_2_-DA; Sigma Aldrich, St. Louis, MO, USA), which is rapidly oxidized to highly fluorescent dichlorofluorescein (DCF) in the presence of ROS. In particular, DCFH_2_-DA and its derivatives are largely used as ROS-sensitive dyes [59] as they diffuse through the plasma membrane into the cytoplasm, where they are deacetylated by intracellular esterase and then oxidized by ROS, producing the green, fluorescent dye DCF. Originally, the oxidation of DCFH_2_-DA to DCF was thought to be specific to H_2_O_2_, but recent evidence has shown that other ROS such as the hydroxyl radical, hydroperoxides, and peroxynitrite can also oxidize DCFH_2_-DA, although with greatly reduced sensitivity compared with that of H_2_O_2_ [60]. Five-week-old Col-8 and *glyI4* leaves were harvested after chemical treatment and *B. cinerea* infection (as in par. 4.9). In particular, for each genotype, per treatment, two leaves from six plants were collected. One leaf was incubated at room temperature in a solution of 20 mM DCFH_2_-DA in 10 mM Tris-HCl (pH 7.4) for 45 min in the dark. As a negative technical control, the remaining leaf was incubated in 10 mM Tris-HCl (pH 7.4) buffer only, under the same conditions. After staining, the samples were washed three times in fresh buffer for 10 min to remove excess fluorophore and mounted on glass slides. Fluorescence was then observed under a LSM 710 confocal microscope (Carl Zeiss Microscopy GmbH, Jena, Germany) with a Plan Neofluar 20× objective and the interfaced software ZEN 2010 (Carl Zeiss Microscopy GmbH, Jena, Germany). Two laser excitations lines were used (488 nm for probe detection and 561 nm for chlorophyll auto-fluorescence). The emission range for the probe was 493–556 nm and for chlorophyll auto-fluorescence it was 585–712 nm. The technical parameters fixed in our acquisition procedure were the pinhole size, at 1 AU (Airy unit); the laser power, at 2%; the digital gain, at 1.0; and 1024 × 1024 frames, with a pixel dwell time of 1.27 us. Images were acquired with a confocal (PMT detectors) an average of 8 times for each laser excitation line. Three independent fields were examined per sample and no z-stack was used. Data were processed using Image J software, version 1.52a (https://imagej.net, accessed on 24 July 2024). The quantification of green fluorescence detected using DCFH_2_-DA in all tested conditions was performed as in [61,62]. Green fluorescence intensity was normalized with the red fluorescence intensity and reported in graphs as the integrated density mean. For each genotype and for each independent experiment, the results from three leaves (from three plants) per treatment have been considered in the analysis.

### 4.9. Statistical Analyses

For the analysis of growth parameters; the determination of chlorophyll, carotenoid, and anthocyanin contents; and RT-qPCR gene expression, a two-way analysis of variance and Šídák’s multiple comparisons test were used to assess the significant differences between the Col-8 and *glyI4* plants among the different treatments. For *B. cinerea* disease resistance bioassays, the significant differences in response to each treatment between Col-8 and *glyI4* were evaluated by the χ^2^ statistical test. A one-way analysis of variance and Dunnett’s multiple comparison test were performed to assess differences in the *B. cinerea* growth rate. A one-way analysis of variance and Tukey’s multiple comparisons test were performed to determine the statistical significance of the chemical treatments within each genotype for the quantification of green fluorescence in the ROS detection assay. Statistical analyses were performed with GraphPad Prism 10.1 (GraphPad Software Inc., San Diego, CA, USA). Descriptions of the samples considered in each independent experiment have been reported specifically in each paragraph of the Materials and Methods. Each independent experiment has been repeated three times. The raw data from the experiments reported in Section 4.3, Section 4.4, Section 4.5 and Section 4.7 and used in the statistical analyses are reported in Table S2.

## Data Availability

The raw data supporting the conclusions of this article will be made available by the authors on request.

## Supplementary Materials

The following supporting information can be downloaded at https://www.mdpi.com/article/10.3390/ijms252212162/s1.
