# Supplementary material for: Arabidopsis GLYI4 Reveals Intriguing Insights into the JA Signaling Pathway and Plant Defense
Source: Int J Mol Sci. 2024 Nov 13;25(22):12162. doi: 10.3390/ijms252212162 (PMC11594653; doi:10.3390/ijms252212162)
Supplement: Supplementary file 1 [file ijms-25-12162-s001.zip › Table S1.pdf]

Table S1. List of primers used in the study for target genes/reference gene and related information

Primer name	5’-3’ sequence	Efficiency (%)	R^2
ACX1 Fw	TCGTCTCAATGAGGAAGAAGC	110.4%	0.995
ACX1 Rv	CCCCTTAATAGCAGGCACAA		
AOC1 Fw	TCATCGAAGCTCTCTTCTTGG	110.5%	0.99
AOC1 Rv	CTTCTTGGGCGTGAAACTTG		
AOS Fw	TACGAATGTCTCCGGTTTGA	105.3%	0.996
AOS Rv	CATTTCACCGGCTTTGACTT		
EIN3 Fw	GCATGTCCACATCGAGACAG	94%	0.997
EIN3 Rv	ACTGAGTTCACTGGCCTTGG		
LOX3 Fw	CGACGGATTCTCCGTACAAC	101.3%	0.98
LOX3 Rv	ATAGGCTTTGCGTCCGTCTA		
MFP2 Fw	CTGCTGCACAGTTAGGCTTG	91.5%	0.993
MFP2 Rv	TTCAAGGGCTTTGGTGAGAC		
OPR3 Fw	CCGGCGGTTTTCTCATCTC	92.9%	0.997
OPR3 Rv	GCTTCCATGCTTCTACTTGT		
JAZ1 Fw	ACTATATTCTACGCCGGGCA	110%	0.993
JAZ1 Rv	CTATTAGCGGTGCCTTTGC		
JAZ3 Fw	AAGAAAGGGTCACGAGCGTA	98.9%	0.989
JAZ3 Rv	GGTTGCAGAGCTGAGAGAAG		
ORA59 Fw	GGGATAAGAGTGTGGCTTGGGACA	107.2%	0.992
ORA59 Rv	TGAGTACTGCGAGGCTGCCTTT		
PDF1.2 Fw	CACCCTTATCTTCGCTGCTCTT	96%	0.997
PDF1.2 Rv	GCCGGTGCGTCGAAAG		
PP2AA3 Fw	TAACGTGGCCAAAATGATGC	106.5%	0.995
PP2AA3 Rv	GTTCTCCACAACCGCTTGGT		
