# Supplementary figures and images for: Arabidopsis GLYI4 Reveals Intriguing Insights into the JA Signaling Pathway and Plant Defense
Source: Int J Mol Sci. 2024 Nov 13;25(22):12162. doi: 10.3390/ijms252212162 (PMC11594653; doi:10.3390/ijms252212162)

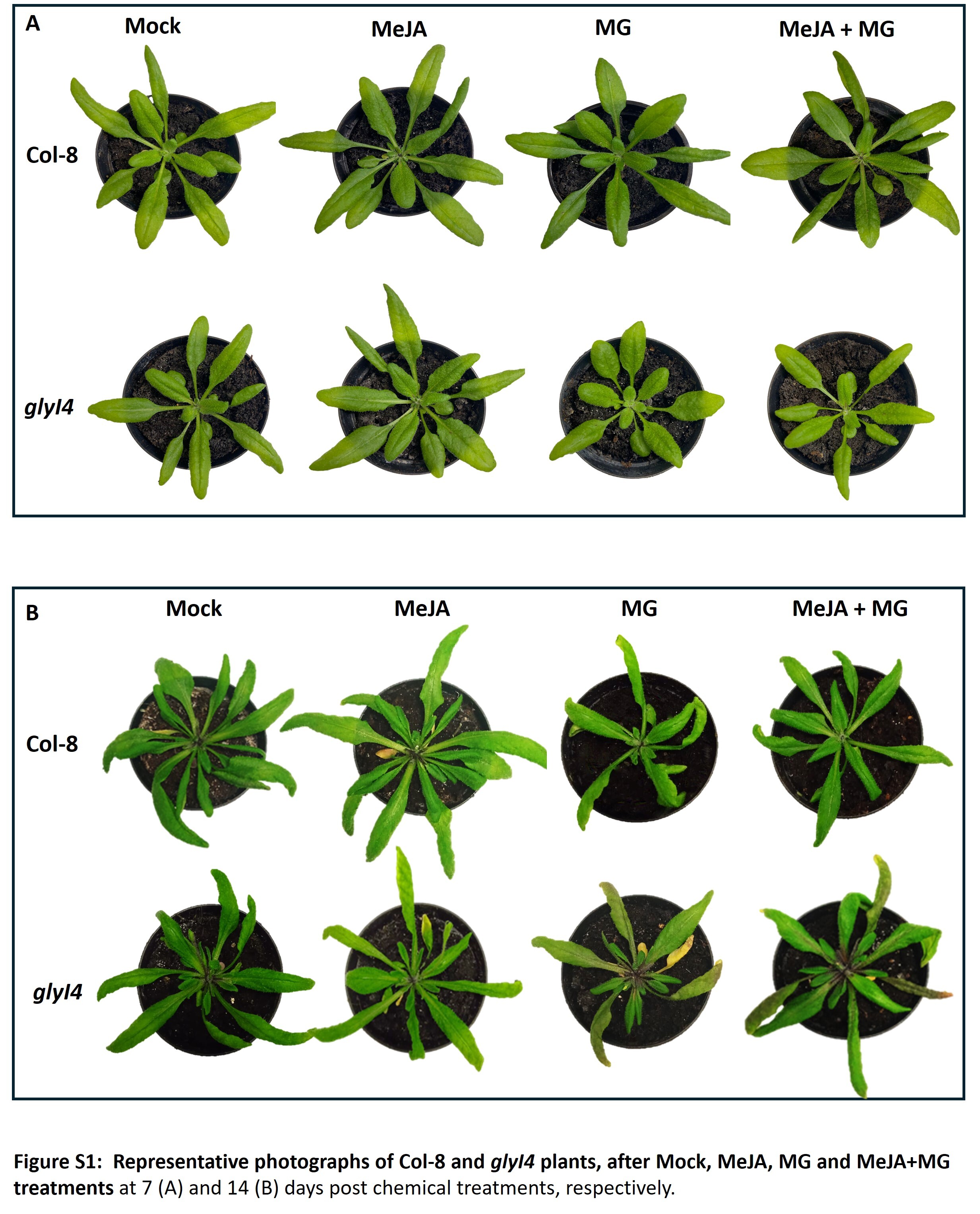

Supplement: Supplementary file 1 [file ijms-25-12162-s001.zip › Figure S1.jpg]

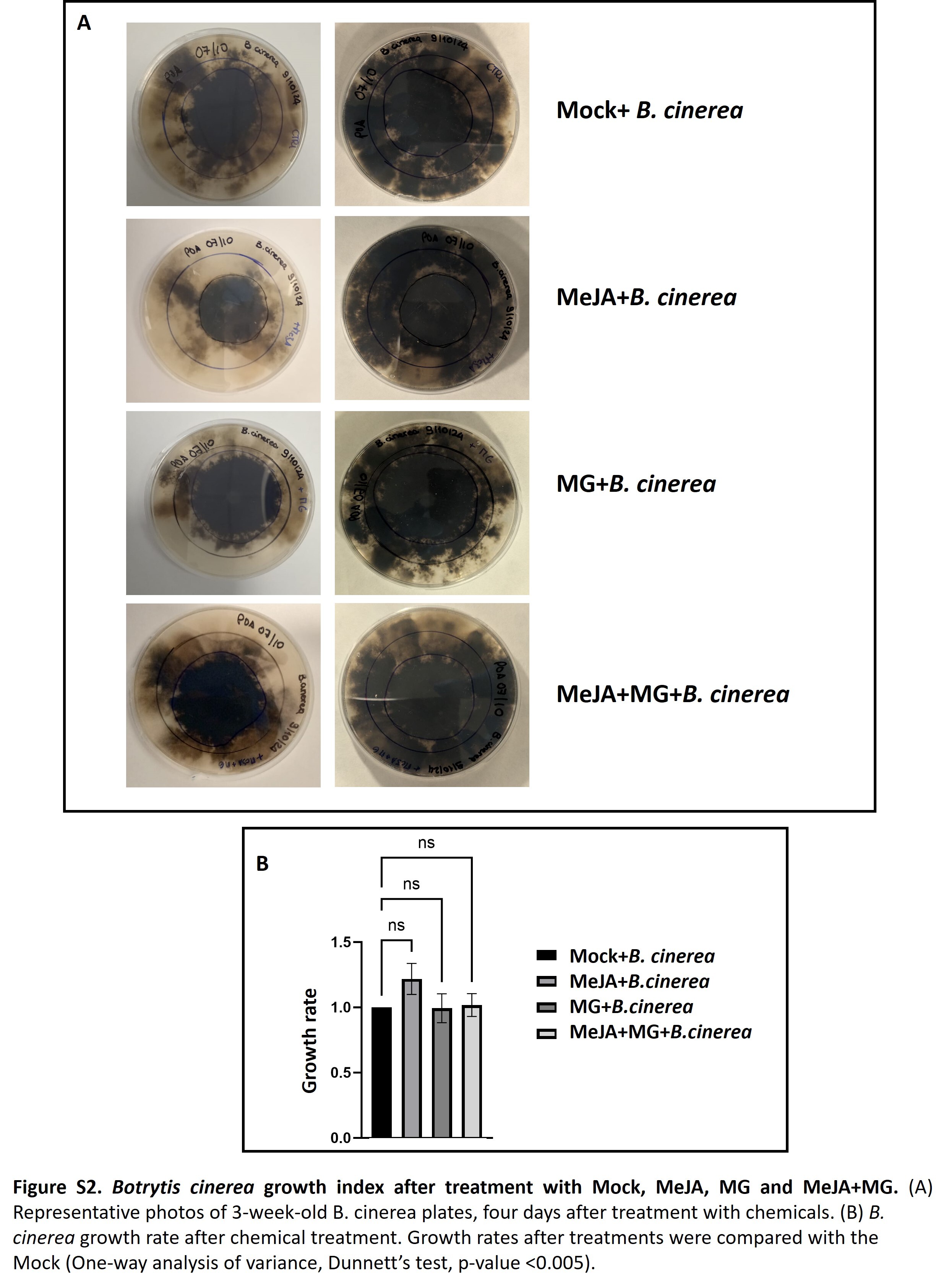

Supplement: Supplementary file 1 [file ijms-25-12162-s001.zip › Figure S2.jpg]
